# Association between nutritional status, body composition, and fitness level of adolescents in physical education in Casablanca, Morocco

**DOI:** 10.3389/fnut.2023.1268369

**Published:** 2023-11-07

**Authors:** Mourad Oukheda, Khawla Bouaouda, Karima Mohtadi, Halima Lebrazi, Abdelfettah Derouiche, Anass Kettani, Rachid Saile, Hassan Taki

**Affiliations:** ^1^Laboratory of Biology and Health, URAC 34, Faculty of Sciences Ben M’sik, Health and BiotechnologyResearch Center, Hassan II University of Casablanca, Casablanca, Morocco; ^2^Mohammed VI Center for Research and Innovation, Rabat, Morocco

**Keywords:** nutritional status, body composition, fitness level, adolescents, physical activity level

## Abstract

**Aim:**

This study aims to analyze and compare dietary intake, as well as to examine the associations between energy intake in terms of macronutrients, body composition, and physical fitness (PF) specifically cardiorespiratory endurance (CE) among a sample of young adolescents aged 15 to 18 years, who participate in physical education and sports sessions in public schools in Casablanca, Morocco.

**Materials and methods:**

A total of 311 participants, including 156 girls and 154 boys, were included in the study. Each participant maintained a food diary for 3 days during the same study week. Additionally, body composition measurements were taken using bioelectrical impedance analysis (BIA). The PF was assessed using the validated mini-Cooper test (6 min).

**Results:**

The results show that the participants had an average total energy intake of 2386.7 ± 492.7 kcal. A significant difference was observed between boys and girls, with average energy intakes of 2468.8 ± 531.1 kcal and 2304.0 ± 437.0 kcal, respectively. These dietary intakes were significantly lower than their needs and nutritional recommendations. The associations of nutritional status, sex, body mass index (BMI) and physical fitness (PF) were tested and a positive correlation was observed following an adequate intake of carbohydrates (CHO) and proteins on Vo_2max_, while a negative association was observed with regard to Body fat for both sexes. Boys exhibit significantly better PF than girls (*p* < 0.01). Obese participants had the lowest PF and an unbalanced nutritional status, the adolescents with a normal weight *p* < 0.01 displayed a high level of PF compared to individuals in other weight categories.

**Conclusion:**

The PF is significantly associated with macronutrient intake status and body composition, especially BMI and BF. The Underweight, overweight, and obese students demonstrated poorer performance in physical fitness indices compared to normal-weight. Adolescents adhering to recommended CHO and protein intake levels tend to exhibit enhanced physical fitness. Implementing strategies to encourage students to maintain a balanced diet and engage in regular physical exercise is essential.

## 1. Introduction

The rapid and simultaneous changes in dietary habits and levels of physical activity have led to a swift increase in obesity and overweight rates, a widely documented phenomenon. This affects not only urban and rural areas of the poorest countries in sub-Saharan Africa and South Asia but also populations in higher-income countries. Although several countries are considering large-scale programs and policy initiatives to address this issue, few of them are effectively implementing substantial efforts to prevent the serious dietary-related problems we currently face (1). In Morocco, the demographic transition it has undergone has been accompanied by shifts in lifestyle, particularly in terms of dietary habits and physical activity (2). This dietary transition is characterized by a shift from traditional food, based on cereals and legumes, to a diet with more animal-derived products and tending to be excessive in relation to the energy needs of a sedentary life. This nutritional evolution has gradually led to a decrease in malnutrition among young children but has also resulted in an increase in overweight and obesity among adolescents and adults, especially in urban areas (3–5).

Overweight and obesity, considered risk factors for non-communicable diseases (NCDs), pose a public health problem on a national scale. They affect both children and adults (6). According to the latest National Survey on Population and Family Health in 2018, the10.8% of children under 5 years are overweight, of which 2.9% suffer from obesity. In comparison, in 2003–2004, the proportion of overweight children was 10.4%, and in 2011, it was 10.7% (7, 8). According to the World Health Organization (WHO) adolescence is a period marked by rapid growth due to puberty, often associated with an increase in physical activity. These characteristics require an individualized approach to this stage of life in many areas, including nutrition and nutritional needs, considering the psychological and behavioral complexity that accompanies it (9, 10). The influence of the nutritional transition, the search for autonomy, and the lack of nutritional education can lead to disruptions in eating habits, which can affect their daily lives and, therefore, their health (11). Adolescents aged 13 to 17 years are also affected by overweight and obesity, with a prevalence of 13.9 and 3%, respectively. Among girls, overweight is much more common than among boys (17.8% versus 10.7%), while for obesity, the difference between the sexes is as follows: 2.7% in boys and 3.3% in girls (7, 8). So, the high prevalence of overweight and obesity persists, and this can be attributed to the rise in sedentary behaviors and the decline in physical activity among adolescents, as noted by Booth et al. (12), In this context, this can affect both body composition and physical fitness level, especially with regard to cardiorespiratory endurance.

Physical fitness (PF) can be regarded as a holistic measure that encompasses numerous facets of bodily functions, including musculoskeletal, cardiorespiratory, hemato-circulatory, psychoneurological, and endocrine-metabolic functions, all of which are involved in the execution of daily physical activities and exercise (13). So, when a validated test of PF is conducted, it genuinely assesses the functioning of all these systems. This is why PF is now recognized as one of the most critical health indicators (13–15). The Cardiorespiratory fitness (CF), sometimes referred to as cardiovascular fitness or maximum aerobic power, indicates a person’s ability to participate in sustained, strenuous activity and their total capability of their circulatory and respiratory systems. The leading single indication of *CF*, according to the World Health Organization, has been the maximum oxygen uptake (Vo_2max_ and expressed in ml.kg^-1^.min^-1^) attained during a progressive maximal activity test until voluntary exhaustion (14). Due to the numerous studies demonstrating the importance of cardiorespiratory endurance as a reliable predictor and key marker of cardiorespiratory health in adolescents and young adults, we have decided to research this aspect of physical fitness (13).

The term of the maximal oxygen uptake was introduced and defined by Hill et al. (16) during the 1920s. The Vo_2max_, as proposed by Hill and Lupton (16), posits the following principles: (1) There exists an upper limit to oxygen uptake, (2) Interindividual differences in Vo_2max_ exist, (3) A high Vo_2max_ is a prerequisite for success in middle and long-distance running, and (4) Vo_2max_ is constrained by the capacity of the cardiorespiratory system to transport oxygen to the muscles.

On the other hand, Body mass index (BMI) is universally considered also a marker of health and is widely used to measure malnutrition, overweight, and obesity (17). It is a widely utilized index for assessing weight status. However, it is important to note that different BMI criteria yield varying outcomes when categorizing underweight, overweight, and obesity, as per the guidelines of the World Health Organization (WHO). On the other hand, body fat measurement has gained prominence in some recent physical fitness assessments and health surveillance programs due to its superior accuracy in diagnosing obesity compared to BMI, as demonstrated in prior studies (18). Body fat is typically assessed using body fat monitoring instruments or skinfold tests. Nonetheless, BMI assessment offers the advantage of requiring only height and weight measurements, making it a straightforward method. Given considerations such as sample size, financial constraints, and time requirements, BMI was selected as the indicator of body composition in the 2018 National Nutrition Survey in Morocco (7). Some studies have revealed a direct correlation between BMI and physical fitness (19). Furthermore, studies have indicated that BMI can effectively indicate the physical fitness of regular college students (19, 20). Moreover, physical fitness correlates positively with physical activity (21). Thus, it holds great importance to track the BMI of high school students for gaining insights into their physical growth and development (22).

In general, a high BMI is typically associated with excess weight or obesity, which may reflect nutritional imbalance, especially excessive calorie intake relative to physical activity. Conversely, a low BMI may indicate insufficient weight, which can also be linked to nutritional issues such as essential nutrient deficiencies (23, 24).

However, it is crucial to note that BMI alone does not provide a comprehensive picture of an individual’s health or nutritional status (25). Other factors, such as body composition, fat distribution, and dietary quality, must also be taken into account to more accurately assess the relationship between nutritional status and health. Some studies have indicated that BMI can be influenced by various factors, as like psychological (26, 27), learning behavior (28), ethnic, Environment and circumstances, such as the COVID-19 Pandemic Period (26, 29) raising questions about its accuracy in reflecting their physical fitness and health (22). It is essential to maintain a balanced intake of macronutrients and micronutrients, along with proper hydration, to ensure an optimal energy supply required for growth, overall health, the prevention of non-communicable diseases (NCDs), meeting the requirements of physical activity, and enhancing athletic performance, and facilitating optimal recovery (30).

It is also important to note that the choice of effective physical activity patterns and proper dietary patterns plays a significant role in the health of adolescents. Associations between physical activity patterns and dietary patterns have been observed in the population of Polish girls. School-going adolescents with the highest adherence to the “school/professional activity” pattern exhibited the highest levels of physical activity and adopted health-promoting dietary behaviors (31–33).

The association between weight status and physical fitness has been investigated in many countries, the research conducted in Western countries found that physical fitness decreased with increasing obesity. A similar trend was also observed in the physical fitness of Chinese children and youth (34). Opting for participation in extracurricular sports could serve as an influential factor in increasing physical activity and promoting an active lifestyle among children. Likewise, minimizing or restricting screen time, especially in children’s bedrooms, may contribute to fostering an active lifestyle (35).

Referring to an analysis of available scientific literature on search engines such as “PubMed,” “Scopus,” and “Science Direct,” there is currently limited data available in Morocco that explores the effect of nutritional intake or nutritional balance on the physical fitness level of adolescents during their schooling years, using validated field tests. This lack of data is surprising given that nutrition has long been recognized as an essential factor for maintaining health and promoting physical and physiological development (36). This motivated us to undertake this work. Firstly, to fill this gap in scientific data, thereby providing a scientific database not only for Morocco but also for Africa, specifically North Africa, and contributed to enriching the broader Mediterranean region as well. Furthermore, our study focuses on determining the adoption of an adequate diet by Moroccan adolescents, with a particular emphasis on the situation in Casablanca. Thus, we aim to establish a diagnosis of the dietary behavior of this age group, which is crucial for assessing their health. Additionally, we strive to gain a better understanding of the consequences of the increasing prevalence of overweight and obesity, as mentioned earlier, on overall health. This concern arises from the possibility that these health issues may have a significant impact on the health of adolescents, as well as on public health in Morocco as a whole.

Therefore, this is the first study to examine the impact of nutritional status, body composition on the physical fitness level, of young Moroccan adolescents enrolled in school, participating in physical education sessions mandated by the educational program in Morocco. It involves a total of 4 h distributed across 3 sessions of physical sports practice per week, with two separate one-hour sessions and one two-hour session grouped together.

The main purpose of this study is to explore possible associations between macronutrient nutritional status, gender, and body mass index (BMI) classes (Normal, Underweight, Overweight, Obesity) on physical fitness (PF), specifically cardiorespiratory endurance (CE) in girls and boys aged 15 to 18 years, who participate in physical education and sports sessions in public schools in Casablanca. Several specific questions can be raised in the context of this study. Do boys exhibit higher physical fitness than girls? Do boys have a better nutritional status than girls? Do individuals with normal weight display better physical fitness? Is excessive consumption of sweet foods more frequent among boys and participants with normal weight? In this context, the following hypotheses have been formulated:

*Hypothesis 1*: Do school-going adolescents meet the recommended energy intake levels, as outlined in the Moroccan Nutrition Guide from the Ministry of Health (2016), designed for adolescents with a moderate level of physical activity (2,100 Kcal + activity-related needs for girls and 2,300 Kcal + activity-related needs for boys), in alignment with established scientific standards?

*Hypothesis 2*: Is having a high level of cardiorespiratory physical fitness associated with adherence to and compliance with the recommended protein intake (1.6 to 2.2 g/kg of body weight) and carbohydrate intake (4 to 8 g/kg of body weight)? In other words, do macronutrient intake levels correlate with physical fitness levels among adolescents?

*Hypothesis 3*: Is a low level of cardiorespiratory physical fitness associated with an abnormal body mass index value and a high percentage of body fat? In other words, are there associations between body composition and physical fitness status.

## 2. Materials and methods

### 2.1. Participants

The study focused on a population of school-going young adolescents, including a total of 310 participants, 156 girls, and 154 boys, aged between 15 and 18 years, who participated in the physical activity prescribed by the educational program of two public high schools in Casablanca. A total of 9 students were excluded from the study, distributed as follows: 2 girls and 2 boys due to incomplete food journals, and 4 girls and 1 boy were excluded due to absence during the physiological test. A total of 301 was analyzed. All subjects provided their consent prior to participating in the survey, in accordance with the authorization obtained from the administration. The present study adhered to the principles outlined in the Helsinki Declaration and received ethical approval from the regional ethics committee N°22/2022.

### 2.2. Design and study sample

After obtaining the necessary approval to conduct the study, two qualifying secondary schools were selected based on their proximity criteria (same geographical area). Subsequently, we introduced ourselves to the directors of the respective schools and explained the objective of the study and the fieldwork tasks to be performed.

To accomplish this, we utilized stratified random sampling for the selection of high schools: in the context of a nutritional study involving school students, this technique was applied to choose two high schools out of four.

**Figure 1:**
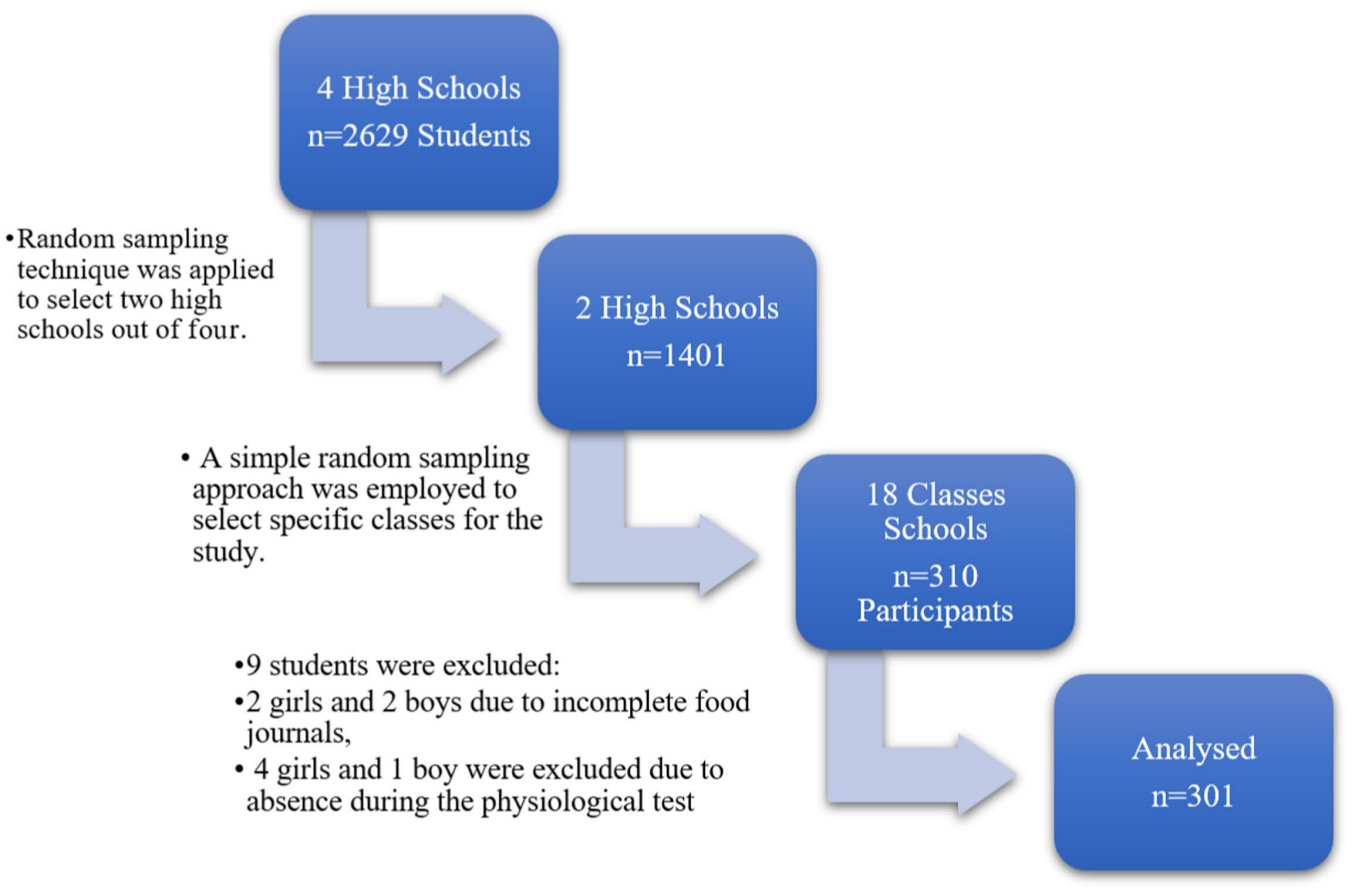
Design and study sample.

Subsequently, within each of the two selected high schools, a simple random sampling approach was employed to select specific classes for the study (Figure 1).

### 2.3. Anthropometric and body composition measurements

All the values were acquired using the In Body 120 bioelectrical impedance analyzer (6–8) developed by In Body Co., Ltd. This analyzer operates at frequencies of 20 and 100 kHz, generating a current of 150 μA (± 50 μA). It provides comprehensive results including weight, BMI, Body muscle mass (BMM), Body fat mass (BFM), and basal metabolism (BM) (37, 38). The subject stood on the footplate with bare feet and held both hand electrodes. It takes 2 min and no specific skills are needed. The precision error of FFM, FM, %BF is <than 2% in 30 subjects (39).

All measurements were conducted over a three-day period, concurrently with the dietary survey. Athletes were instructed to remove their shoes, while height measurements of the subjects were taken using a stadiometer with a precision of ±0.1 mm for utmost accuracy (Portable Statimeter-Seca 225, Hamburg, Germany).

We adopted the reference values from the World Health Organization for assessing the body profile of adolescents aged 10–19 years (40). These reference values were used to determine the prevalence of underweight, overweight, and obesity among the adolescent subjects, taking into account each age group and gender.

### 2.4. Dietary intake data collection

The dietary contribution of adolescents was assessed using 3-day food diaries (41–43). The 3-day food diary method is a widely used tool for dietary assessment, where individuals record their food consumption over a period of three consecutive days. It offers several advantages, including the provision of detailed dietary information, reliance on self-reporting, cost-effectiveness, and suitability for longitudinal studies. However, it has limitations, such as reliance on memory recall, the potential for social desirability bias, and a limited assessment period. Despite these drawbacks, the 3-day food diary remains a simple yet valuable tool in the field of nutritional research (44, 45). After the training session on the objective of this nutritional study, food recording journals were distributed to the students. These journals were provided with detailed instructions and a practical example to prevent incorrect or inaccurate journaling. During the process, participants are instructed to use common household measurements such as spoons, glasses, and dishes to provide the most accurate estimation of the amounts consumed. They are also encouraged to photograph their meals whenever possible. Additionally, participants were provided with the researchers’ contact information to reach out to the research team in case of any questions or concerns regarding the food recording process. We used the Moroccan and OMS recommendations to compare macronutrients intake (6, 7, 46).

### 2.5. Dietary intake analysis

The analysis of dietary intake was carried out using the Nutrilog 3.30 professional software (47), which utilized the Ciqual 2020 food composition database. This comprehensive database offered detailed information on the nutritional content of various components such as carbohydrates, sugars, proteins, fat, fatty acids, salt, vitamins, minerals, and energy values of foods. To ensure the highest level of accuracy in the results, the analysis was conducted by a specialized nutrition researcher from our laboratory.

### 2.6. Performance assessment

Considering the specific characteristics of the target age group, we assessed the participants’ athletic performance using the mini-Cooper test. This modified version draws its inspiration from the original Cooper test, which was originally developed by Cooper (48, 49) for assessing physical fitness and cardiorespiratory endurance. The core objective of this test, in its original form, is to measure how far an individual can run within a 12-min time period. It is crucial to emphasize the importance of maintaining an appropriate pace during the test, as starting with a pace too close to an all-out sprint may impede the achievement of maximum distance. The accuracy of the Mini-Cooper test in diagnosing physical fitness has been validated (49). This test is primarily designed to assess cardiorespiratory endurance by measuring the distance covered during a maximal effort. It provides a quick evaluation of endurance and is relatively straightforward to administer (48, 50).

The mini test cooper involves running the greatest possible distance at a sustained pace for 6 min. It allows for the estimation of Maximal Aerobic Speed or Maximal Aerobic Velocity (MAS or MAV) (Km/h) (51, 52), and to estimate the maximal volume of oxygen consumed Vo_2max_ (ml/kg/min) (53). According to the following equations:


$$\begin{array}{c} MAS=\left(\mathrm{Distance}\;\mathrm{traveled}\;\mathrm{in}\;\mathrm{meters}/100\right)\;\mathrm{km}/h.V_{O2\;\mathrm{MAX}}\\ =MAS\;x\;3.5=\mathrm{ml}/\mathrm{kg}/mn \end{array}$$

The participant’s maximum distance covered during the test was measured. A qualified physical education teacher and the research team were present throughout the test, ensuring a safe process.

After completing the test, the participants were allowed to continue running at a lower intensity for a certain period of time to cool down.

### 2.7. Statistical analysis

The analysis of the collected data was performed using the Statistical Package for the Social Sciences (SPSS) software (IBM, SPSS Statistics, Version 27, Chicago IL). Descriptive statistics were determined: minimum (min) and maximum (max) value, mean value (x) and standard deviation (SD). The normal distribution of differences between data pairs was verified with Shapiro–Wilk tests (*p* > 0.02 for all variables). Additionally, the categorical variables were tested using the Chi2 test; The differences between genders (girls and boys), also between variables and recommended values were compared using the student’s *t*-test. The ANOVA test (with *post hoc* tests such as the Tukey test and Dunnett’s C) was used to determine the relationship between weight categories and cardiorespiratory physical fitness levels, the test Pearson’s correlation coefficient (*r*) was used to examine correlations between the selected variables. Statistically significant differences between the studied groups or association between variables were confirmed if the *p*-value was less than 0.05.

## 3. Results

To achieve the main objective of this study, which is to explore potential correlations between nutritional status, body composition, and cardiorespiratory physical fitness, we followed a methodical plan to ensure a logical progression of the analysis, moving from the general to the specific. First, we present the anthropometric and body composition characteristics of the sample, allowing us to better grasp the weight status of the adolescents under investigation and make comparisons between the two genders (females and males). Subsequently, we conducted an assessment of energy intake and macronutrient consumption. This falls within a comparative framework with prevailing nutritional recommendations, with the aim of determining whether adolescents adhere to these guidelines. Moving forward, we compared the values of dietary intake and Vo_2max_ relative to body composition while distinguishing between the two genders in order to identify potential significant differences.

The final section, titled “3.4 Exploratory Study,” seeks to investigate and present potential correlations between energy intake and various variables associated with body composition, such as body mass index (BMI) and body fat mass. Additionally, we examine the relationships existing between Vo_2max_ and these same variables, as well as with macronutrient intake, including carbohydrates, proteins, and fats. This comprehensive approach aims to provide an in-depth analysis of potential associations between nutritional aspects, body composition, and cardiorespiratory fitness.

### 3.1. Characteristics and weight status of the sample

A total of 301 students from a public high school in Casablanca were analyzed Table 1, comprising 150 girls and 151 boys, with an average age of 16 ± 1 year. The *t*-test revealed no significant difference between the two sexes concerning age (*p*-value > 0.05). However, significant differences were observed in height (mean of 166.3 ± 9.0 cm for girls and 172.2 ± 7.6 cm for boys) and weight (mean of 61.3 ± 11.1 kg for the overall group, 59.1 ± 10.2 kg for girls, and 63.4 ± 11.5 kg for boys) with *p*-values <0.01. Similarly, there were significant differences in body mass index (BMI) with an overall mean of 22.2 ± 3.7 of all genders, the 21.4 ± 3.6 for boys, and 23.0 ± 3.6 for girls. Basal metabolic rate (BMR) was also significantly different, with mean values of 1572.2 ± 153.9 Kcal for girls and 1690.5 ± 174.1 Kcal for boys. These results were obtained using a bioelectrical impedance analyzer, the In Body 120.

**Table 1:** The anthropometry characteristics of the sample of the studied population.

	Gender												*p*-value
	Females				Males				Total				
	Mean	SD	Min	Max	Mean	SD	Min	Max	Mean	SD	Min	Max	
Age	16	1	15	18	16	1	15	18	16	1	15	18	>0.01
Height (cm)	160.4	5.8	148.0	177.0	172.2	7.6	153.0	191.0	166.3	9.0	148.0	191.0	<0.01
Weight (Kg)	59.1	10.2	38.5	95.0	63.4	11.5	37.0	103.0	61.3	11.1	37.0	103.0	<0.01
BMI	23.0	3.6	15.4	33.7	21.4	3.6	15.3	36.9	22.2	3.7	15.3	36.9	<0.01
BM Kcal	1572.2	153.9	1269.8	2091.0	1690.5	174.1	1243.8	2280.8	1631.5	174.4	1243.8	2280.8	<0.01

SD, standard deviation; BM, body mass; BMI, body mass index; BM, Basal metabolic; *p*-value significative value < 0.05.

**Figure 2:**
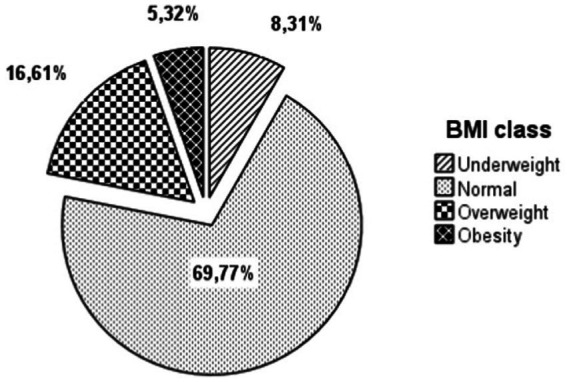
Distribution of the BMI groups of the studied population.

**Figure 3:**
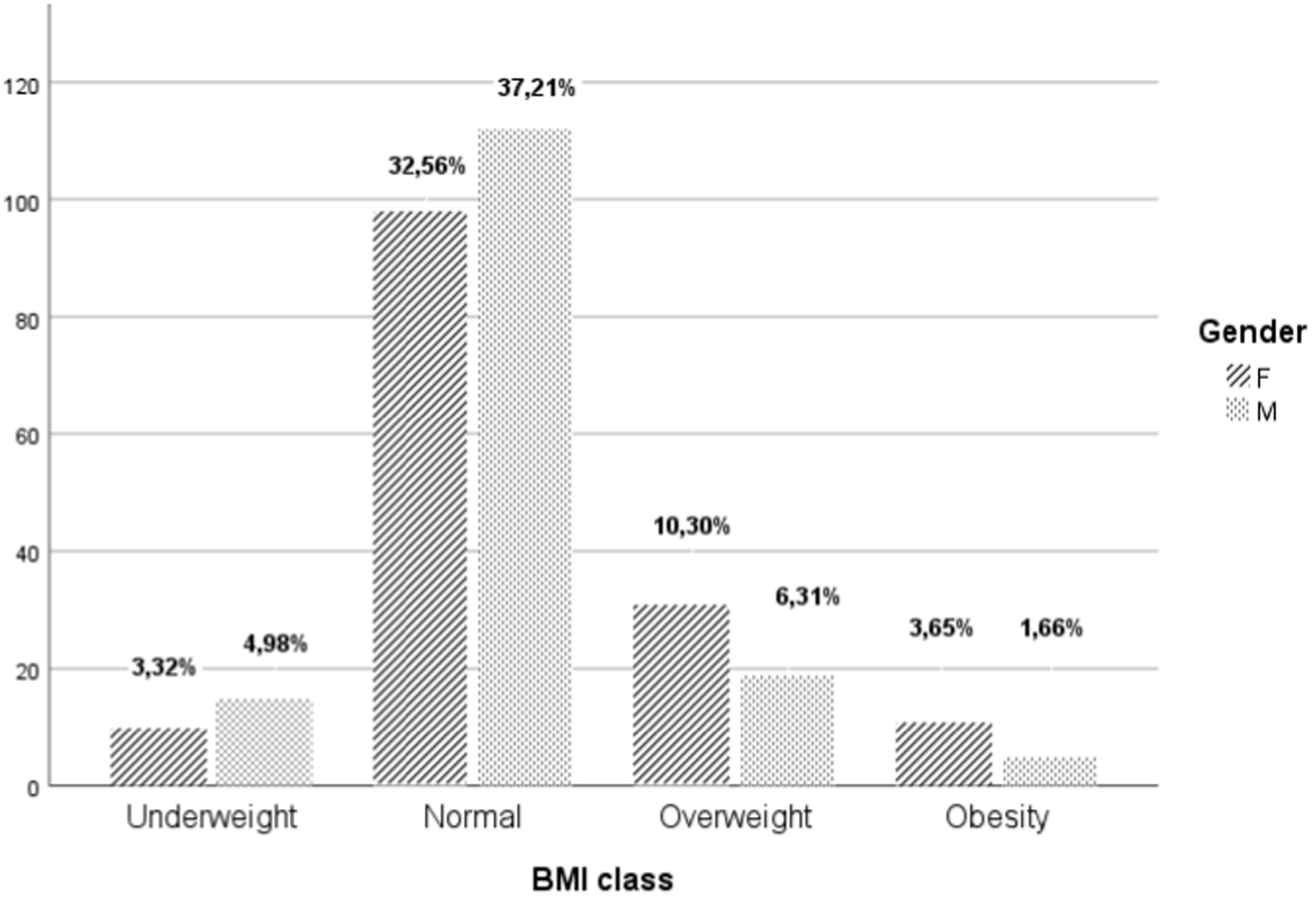
Distribution of the BMI groups of the studied population by gender.

The analysis of the body profile shows that Table 2. 30.23% of the students have abnormal body composition, distributed as follows: 16.61% are overweight, with a higher prevalence in females compared to males (10.30% of girls and 6.31% of boys). Additionally, obesity is present in 5.32% of the students, with a significantly higher occurrence in females compared to males (3.65% of girls and 1.66% of boys). Furthermore, 8.31% of the students are undernourished, with a slightly higher percentage in boys compared to girls (4.98% of boys and 3.32% of girls). The majority of students, 69.77%, have a normal body composition Figures 2, 3.

**Table 2:** The BMI class of the studied students.

		Gender				Total	
		F		M			
		(*n* = 150)	% of total	(*n* = 151)	% of total	(*n* = 301)	% of total
BMI class	Underweight	10	3.3%	15	5.0%	25	8.3%
	Normal	98	32.6%	112	37.2%	210	69.8%
	Overweight	31	10.3%	19	6.3%	50	16.6%
	Obesity	11	3.7%	5	1.7%	16	5.3%
Total		150	49.8%	151	50.2%	301	100.0%

BMI, body mass index.

### 3.2. Energy and dietary intake evaluation

#### 3.2.1. Energy intake

The Shapiro–Wilk test revealed that the data pertaining to the total energy intake follows a normal distribution (*p*-value > 0.02 was observed). These young students receive an average dietary intake Table 3 of energy 2,387 ± 492 calories. A significant difference was observed between boys and girls regarding the energy intake (*p*-value < 0.01). Indeed, boys’ energy intake is higher than that of girls, with an average of 2,486 ± 531 calories for boys and 2304.0 ± 437 calories for girls.

**Table 3:** Values of daily energy and macro nutrient intake values of all participants.

	Gender												*p*-value
	F				M				Total				
	Mean	SD	Min	Max	Mean	SD	Min	Max	Mean	SD	Min	Max	
TEI (Kcal)	2304.0	437.0	1070.3	3581.5	2468.8	531.1	1102.6	3998.4	2386.7	492.7	1070.3	3998.4	<0.01
CHO%	52.1	8.0	31.8	60.4	54.9	6.1	32.7	60.4	53.5	7.2	31.8	60.4	<0.01
CHO	5.1	0.9	3.0	6.0	5.3	0.8	3.0	6.0	5.2	0.9	3.0	6.0	<0.01
PRO %	13.0	3.4	5.5	17.2	13.6	3.2	1.2	17.1	13.3	3.3	1.2	17.2	>0.05
PRO	0.9	0.4	0.2	1.3	0.9	0.4	0.2	1.2	0.9	0.4	0.2	1.3	>0.05
FAT %	34.0	10.9	24.6	61.0	30.5	8.6	24.3	62.5	32.2	10.0	24.3	62.5	<0.01
FAT	1.5	0.5	1.0	2.5	1.3	0.4	1.0	2.4	1.4	0.4	1.0	2.5	<0.01

Total energy intake (Kcal), Ratio of CHO%, CHO g/kg BM, Ratio of PRO %, PRO g/kg BM, Ration of FAT %, FAT g/kg BM, *p*-value significative value < 0.05.

**Figure 4:**
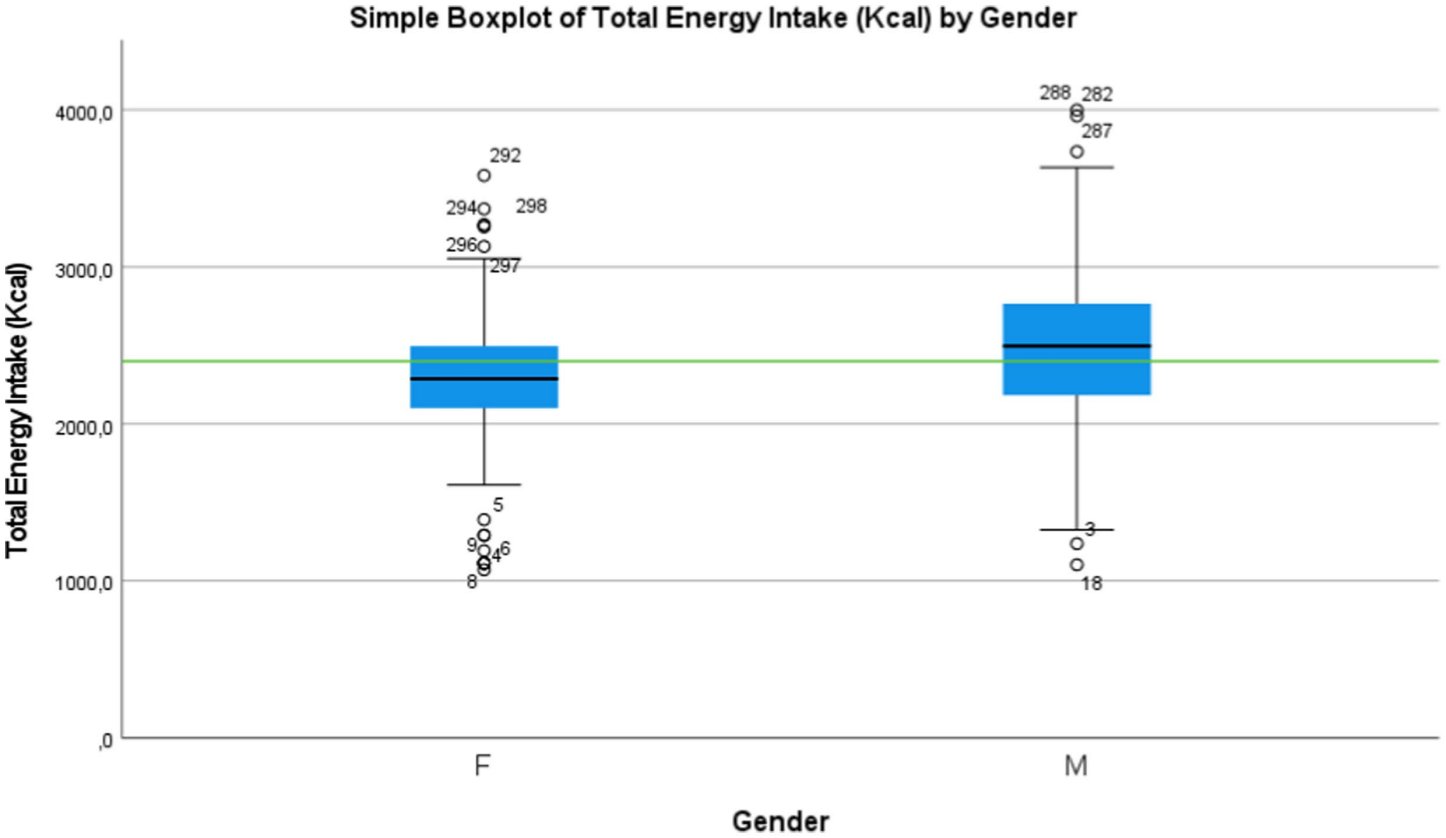
Distribution of the Energy Intake and Moroccan recommendation (green line).

This calorie distribution is as follows: 53.5% comes from carbohydrates, 14.3% from proteins, and 32.2% from fats in terms of proportions. However, the proportion of fat intake is significantly higher in girls (34.0% ± 10.9) compared to boys (30.0% ± 10.9) (*p*-value < 0.01) regarding hypothesis 1 (H1): the results indicate that most girls have energy intake below the recommended levels to meet the demands of this physical effort, while nearly half of the boys have energy intake that is almost adequate Figure 4. This difference in energy intake between the two groups was statistically confirmed using the student’s *t*-test.

#### 3.2.2. Macronutrient’s intake

As presented in the previous Table 3, the average carbohydrate intake per body weight per day for boys (5.3 ± 0.8 g/kg/day) is significantly higher than that of girls (5.1 ± 0.9 g/kg/day) (*p-*value < 0.01). Likewise, a significant difference was observed in lipid intake, with girls having an average intake of 1.5 g/kg/day, which is significantly higher than boys’ intake of 1.3 g/kg/day. This lipid trend explains the higher lipid percentage in girls, reaching 34%, compared to 30.5% in boys.

The study also found that the contribution of fats to the total energy intake was 34.5 ± 1.3%. This value slightly exceeds the upper recommended limit of 30% for fat intake. Additionally, the cholesterol value also exceeds the upper recommended limit.

However, no significant difference was observed in terms of protein intake, with 0.9 ± 0.3 for girls and 0.9 ± 0.4 for boys (*p-*value > 0.05). Based on these all results, hypothesis 2 (H2) has been confirmed.

### 3.3. Comparison of values of dietary intake, body composition, and Vo_2max_

The Shapiro–Wilk test revealed that the data pertaining to Body Composition (BMI, Body Fat) and also Vo_2max_ follow a normal distribution (*p*-value > 0.02 was observed). Body Composition and Vo_2max_. To explore the relationships further, Pearson’s correlation coefficient (r) was used to determine correlations, and statistically significant differences between the studied groups or associations between variables were confirmed if the *p*-value was less than 0.05 (Table 4).

**Table 4:** Comparison of values of, body mass index, and physical fitness in Vo_2max_ and MAS (km/h).

		Gender				*p-*value T-test
		F		M		
		MAS (km/h)	Vo_2max_ (mL/min/kg)	MAS (km/h)	Vo_2max_ (mL/min/kg)	
		Mean ± SD	Mean ± SD	Mean ± SD	Mean ± SD	
BMI class	Underweight	8.6 ± 0.2	30.3 ± 0.8	10.4 ± 0.7	36.3 ± 2.5	<0.01
	Normal	10.4 ± 0.9	36.5 ± 3.1	12.1 ± 0.9	42.5 ± 3.0	<0.01
	Overweight	8.9 ± 0.4	31.3 ± 1.5	10.6 ± 0.5	37.0 ± 1.8	<0.01
	Obesity	8.1 ± 0.2	28.3 ± 0.7	9.6 ± 0.5	33.6 ± 1.9	<0.01

BMI: body mass index; Vo_2max_: Volume maximal of oxygen uptake; MAS: Maximal Aerobic Speed.

### 3.4. Exploratory study

#### 3.4.1. Correlation between energy intake and body composition

**Figure 5:**
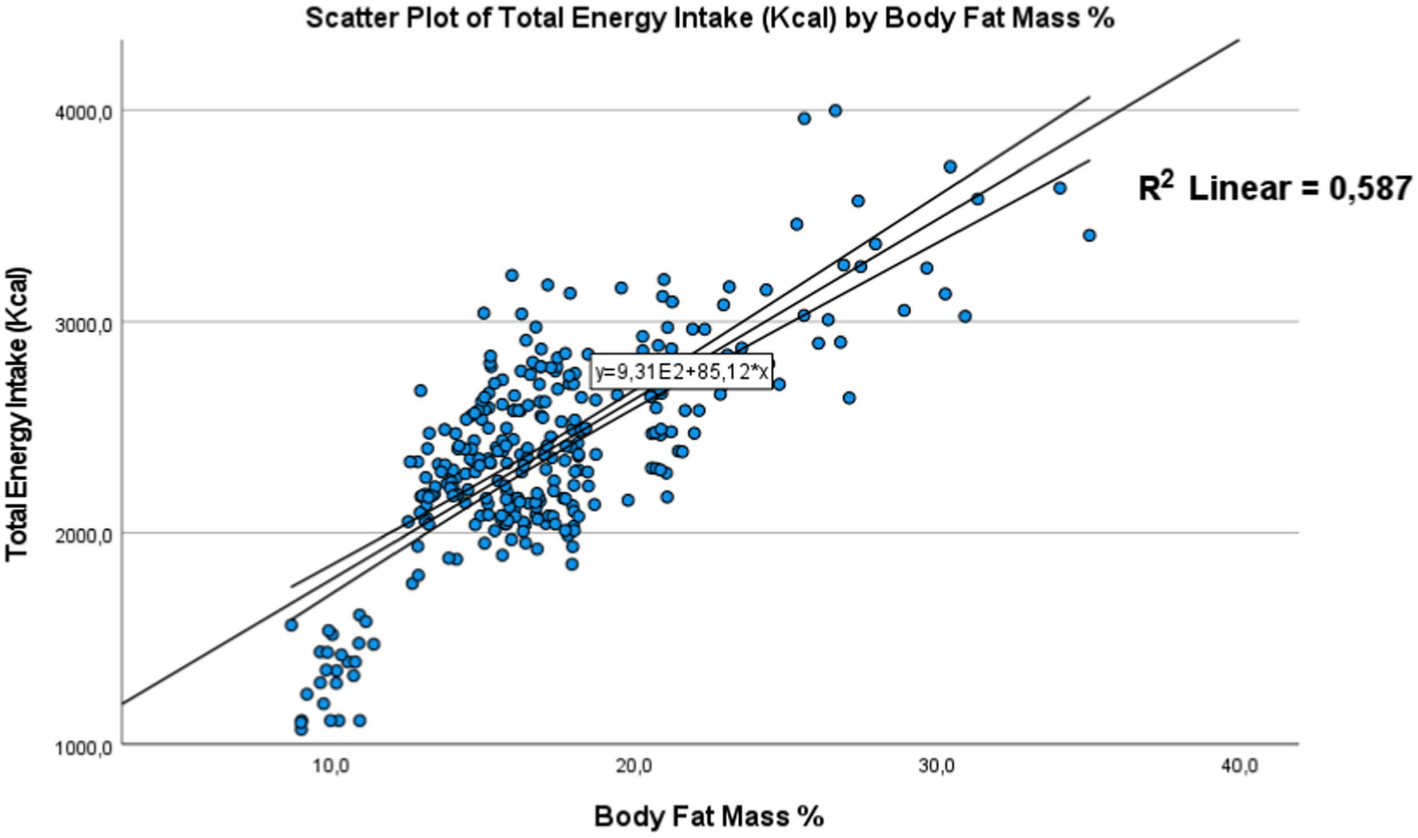
Correlation between energy intake and body fat mass the dots represent individual participants (*n* = 301). The area is for the 95% confidence interval.

**Figure 6:**
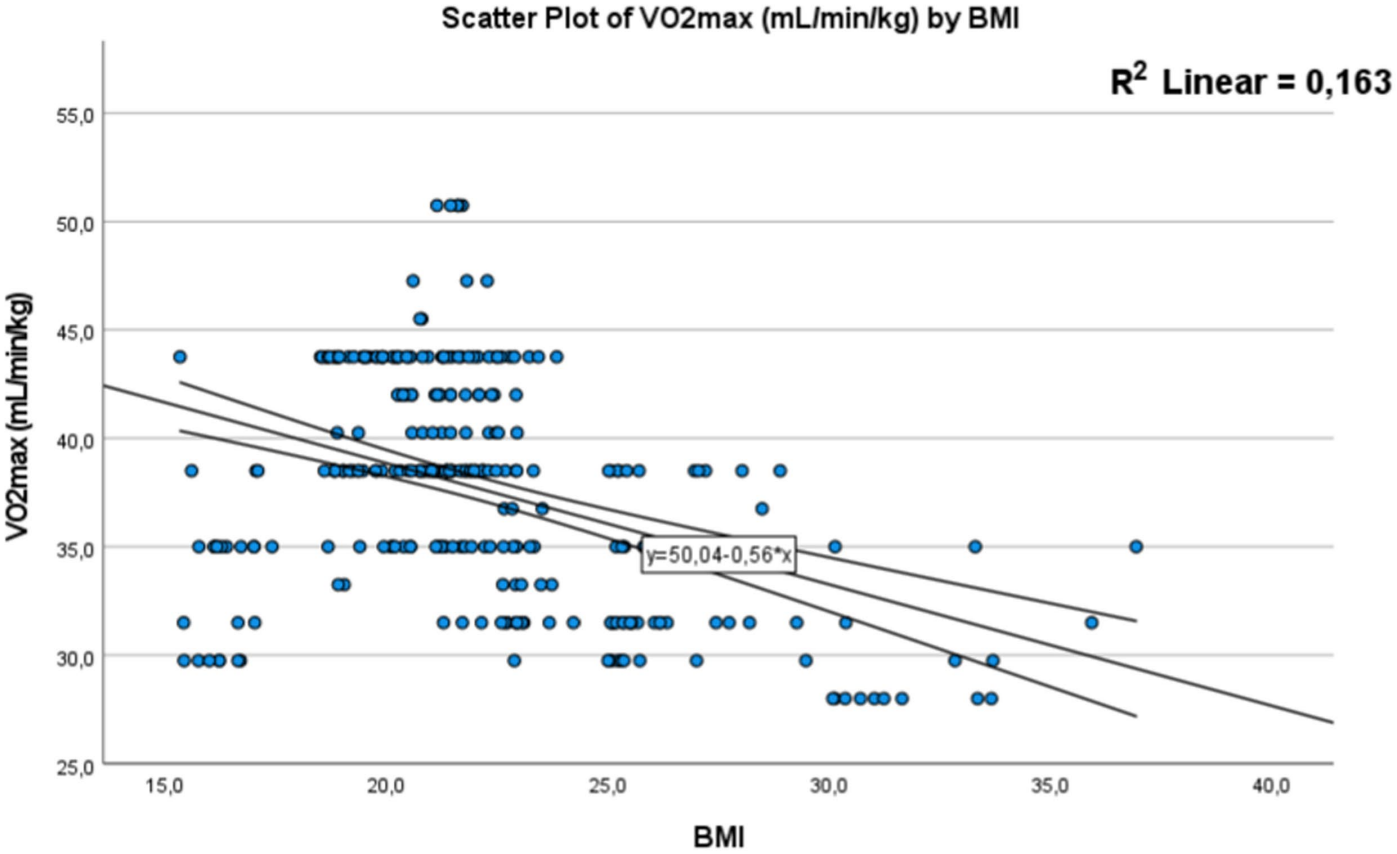
Correlation between (BMI) body mass index and Vo_2max_. The dots represent individual participants (*n* = 301). The area is for the 95% confidence interval.

We employed Pearson’s correlation coefficient (r) to assess the connection between energy intake and body composition. Specifically, we investigated the influence on body fat percentage and lean BMI. The results depicted in Figures 5, 6 indicate that no significant correlation was found between these variables (*p* > 0.05). The dots represent individual participants (*n* = 301). The area is for the 95% confidence interval.

##### 3.4.1.1. Correlation between energy intake and BMI

**Figure 7:**
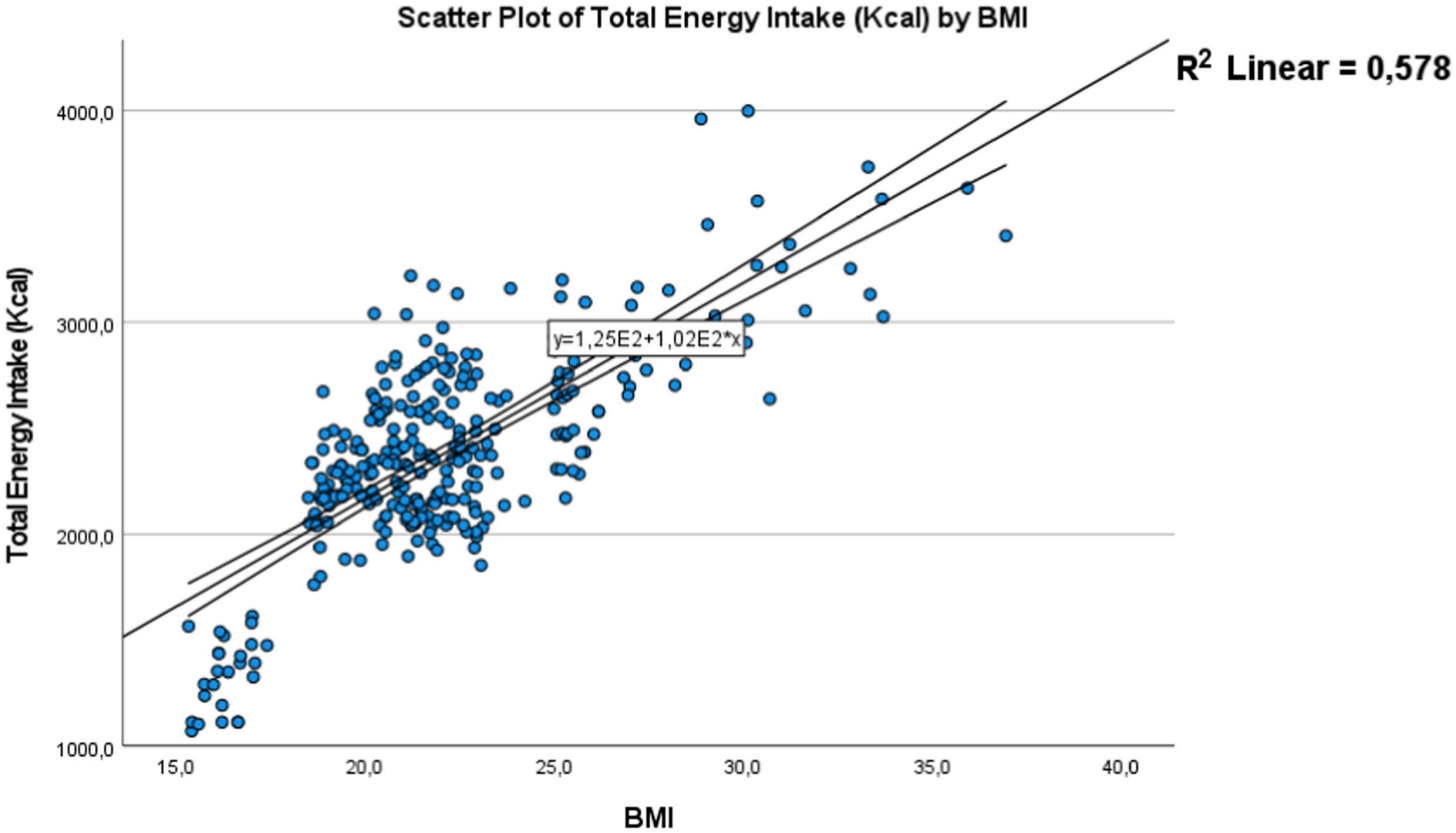
Correlation between energy intake and body mass index the dots represent individual participants (*n* = 301). The area is for the 95% confidence interval.

Using Pearson’s correlation, we found a significant correlation between energy intake and body mass index Figure 7, with a *p*-value <0.01 and a correlation coefficient of 0.76, along with a coefficient of determination (*R*^2^) of 0.57. It appears that adolescents with higher energy intake tend to have a higher body mass index. These results suggest a notable association between diet and body composition among the adolescents in the studied sample.

##### 3.4.1.2. Correlation between energy intake and body fat mass (BFM)

Similarly, to the results obtained for body mass index, the body fat percentage also shows a positive correlation Figure 5. A *p*-value < 0.01 was obtained, with a correlation coefficient (*R*) of 0.766 and a coefficient of determination (*R*^2^) of 0.587, confirming a significant link between the high energy intake received by these adolescents and their tendency to have a higher body fat percentage. This correlation is particularly pronounced in girls compared to boys. These results suggest that adolescents with a higher body fat percentage tend to consume more energy-rich foods.

#### 3.4.2. Vo_2max_ and body composition

In this section, our objective was to determine the potential correlations that could positively or negatively impact Vo_2max_ as a scientific tool for assessing physical fitness, in relation to body composition, specifically body mass index (BMI) and body fat percentage. We also sought to gain an understanding of the impact of dietary intake and eating behavior of these students on Vo_2max_.

For this reason, we chose to adopt the Pearson test to assess the relationship between Vo_2max_ and BMI on one hand, and body fat percentage on the other. Additionally, to better comprehend the relationship between different weight categories and Vo_2max_, we opted for an ANOVA (Analysis of Variance) test and created a graphical representation to visualize the variability of this variable among these groups.

These analyzes will provide us with a clear picture of the connections between individuals’ weight status, their Vo_2max_, and the influence of body composition and eating behavior on this measure of physical fitness.

##### 3.4.2.1. Correlation between Vo_2max_ and BMI and body fat mass

A significant correlation with *p* < 0.01 was found between body mass index (BMI) and Vo2max level Figure 6. with a negative correlation coefficient of −0.40 (*R*^2^ = 0.163). This finding indicates that an increase in BMI is associated with a decrease in physical fitness performance.

**Figure 8:**
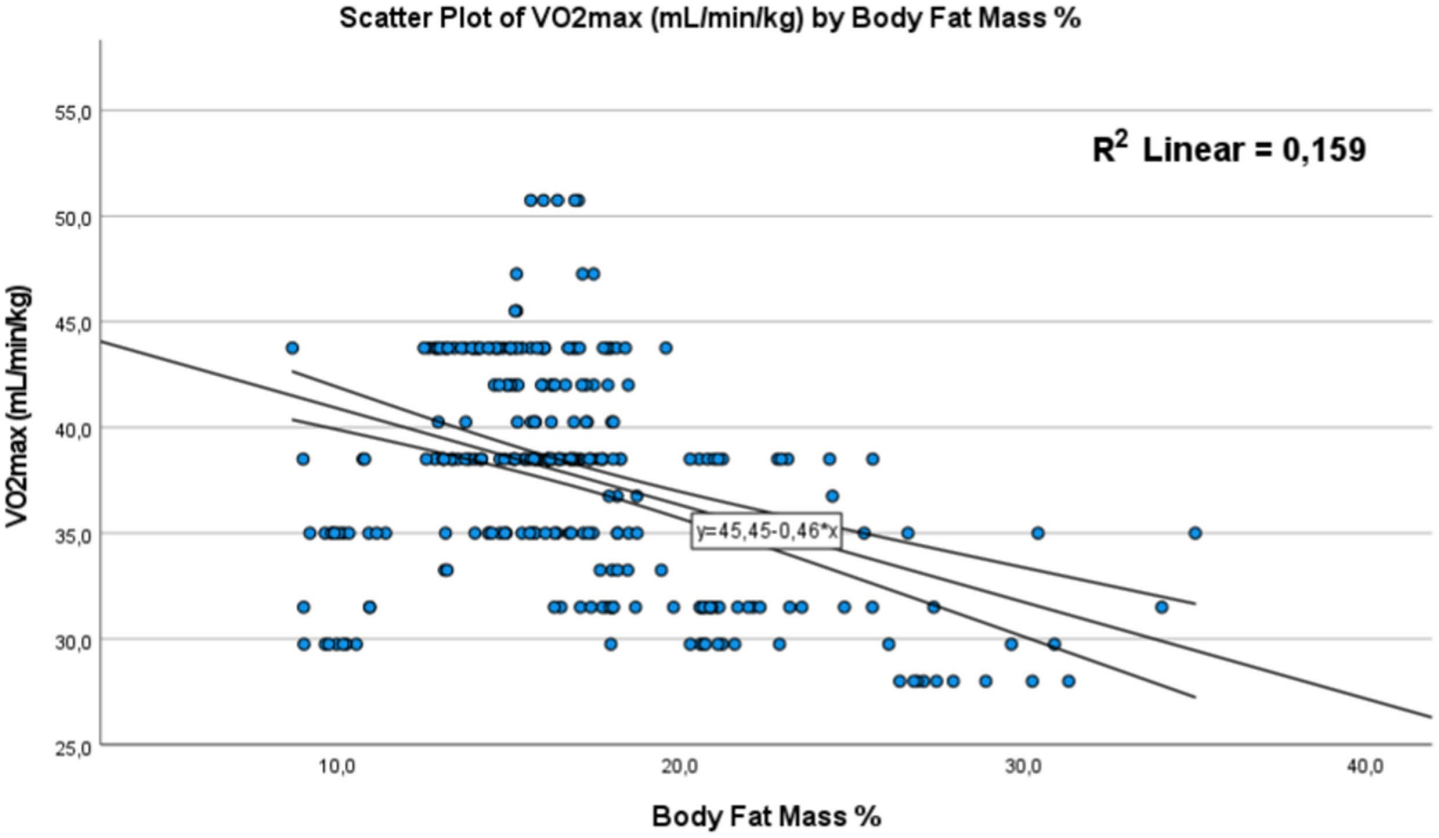
Correlation between the (BFM) body fat mass % and Vo_2max_. The dots represent individual participants (*n* = 301). The area is for the 95% confidence interval.

Similarly, body fat mass also showed a significant negative correlation with physical fitness performance Figure 8. with a *p*-value < 0.01 and a correlation coefficient of −0.39 (*R*^2^ = 0.159). This suggests that an increase in body fat percentage is linked to a reduction in physical performance.

In summary, these results highlight a negative association between body mass index, body fat mass, and Vo_2max_ level, meaning that as BMI or body fat percentage increases, physical fitness performance decreases hypothesis 3 (H3).

##### 3.4.2.2. Vo_2max_ and BMI class of participants

**Figure 9:**
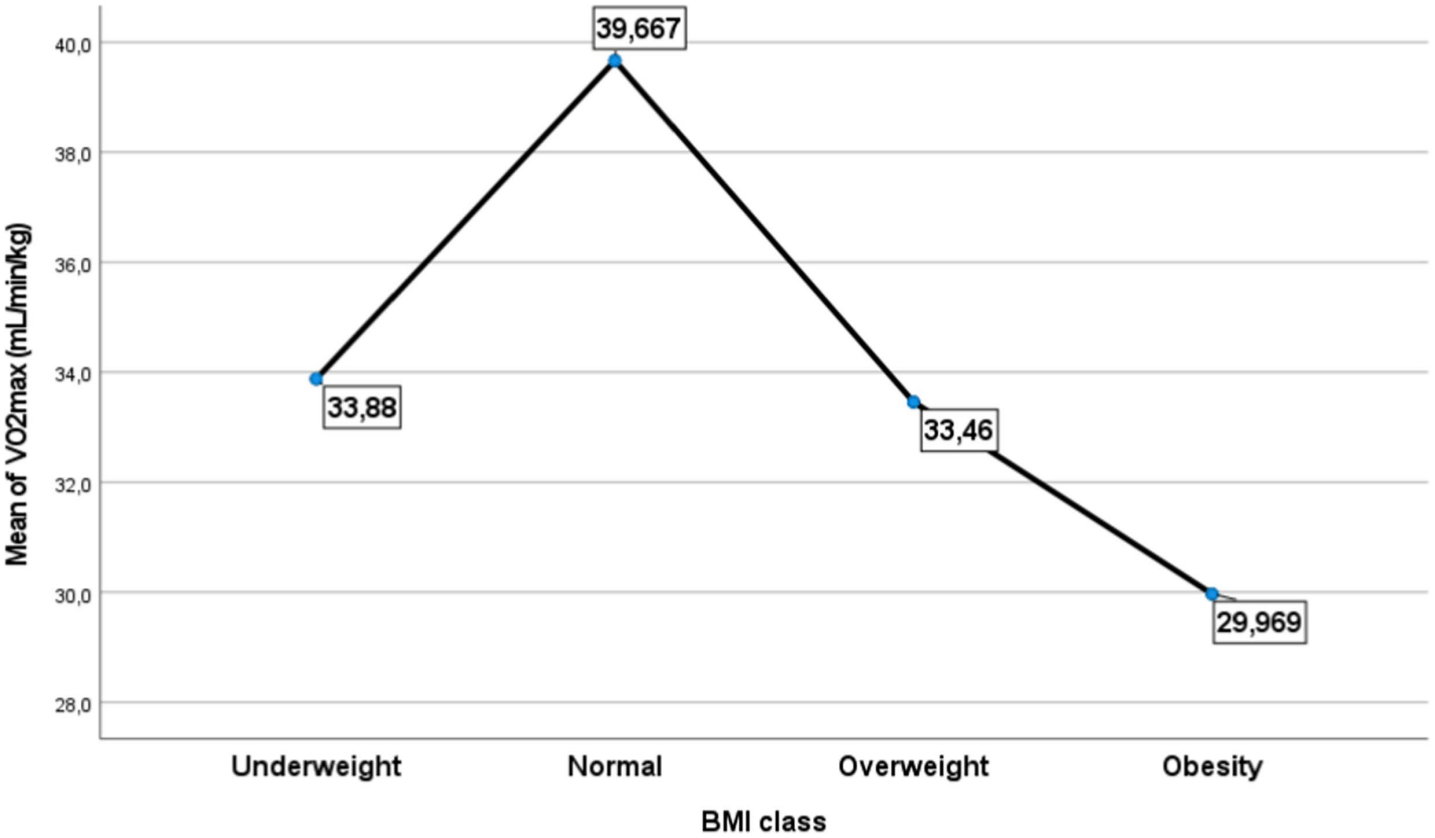
Distribution the mean of Vo_2max_ by class of body mass index.

The recent results have been validated by the analysis of variance (ANOVA), highlighting a significant relationship between Vo_2max_ levels and different weight categories within the studied sample Figure 9. The data distribution, illustrated by the plot, confirms that adolescents with a normal weight were predominant (69.77%; *p* < 0.01), with a higher proportion of boys compared to girls, and all of them exhibited a high level of physical fitness compared to other weight groups. Conversely, obese individuals showed the lowest performance compared to overweight and lean individuals.

#### 3.4.3. Vo_2max,_ and macronutrient

##### 3.4.3.1. Correlation between Vo_2max_ and carbohydrates in g/kg of body mass in day

**Figure 10:**
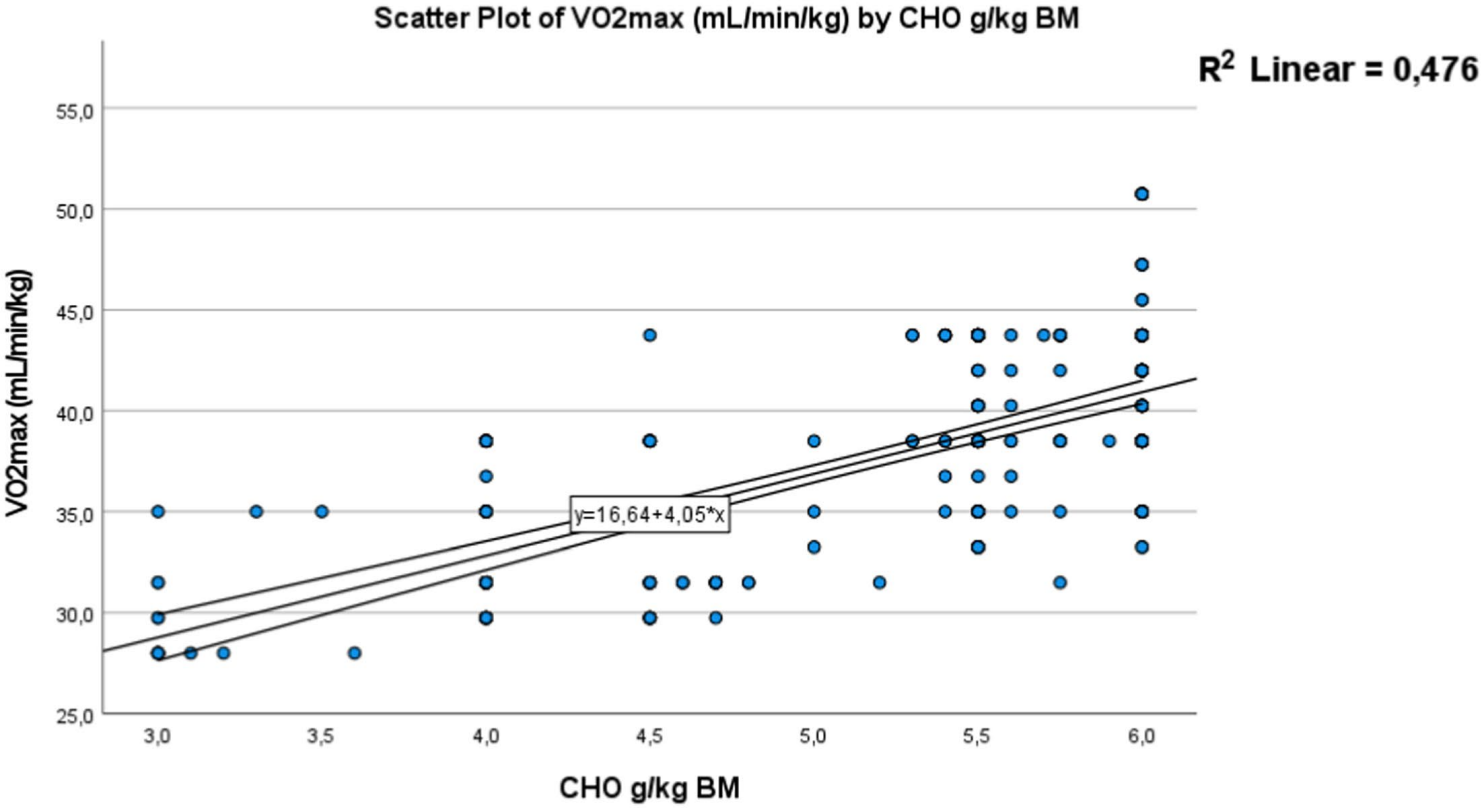
Correlation between (CHO) the carbohydrate’s intake and Vo_2max_. The dots represent individual participants (*n* = 301). The area is for the 95% confidence interval.

**Figure 11:**
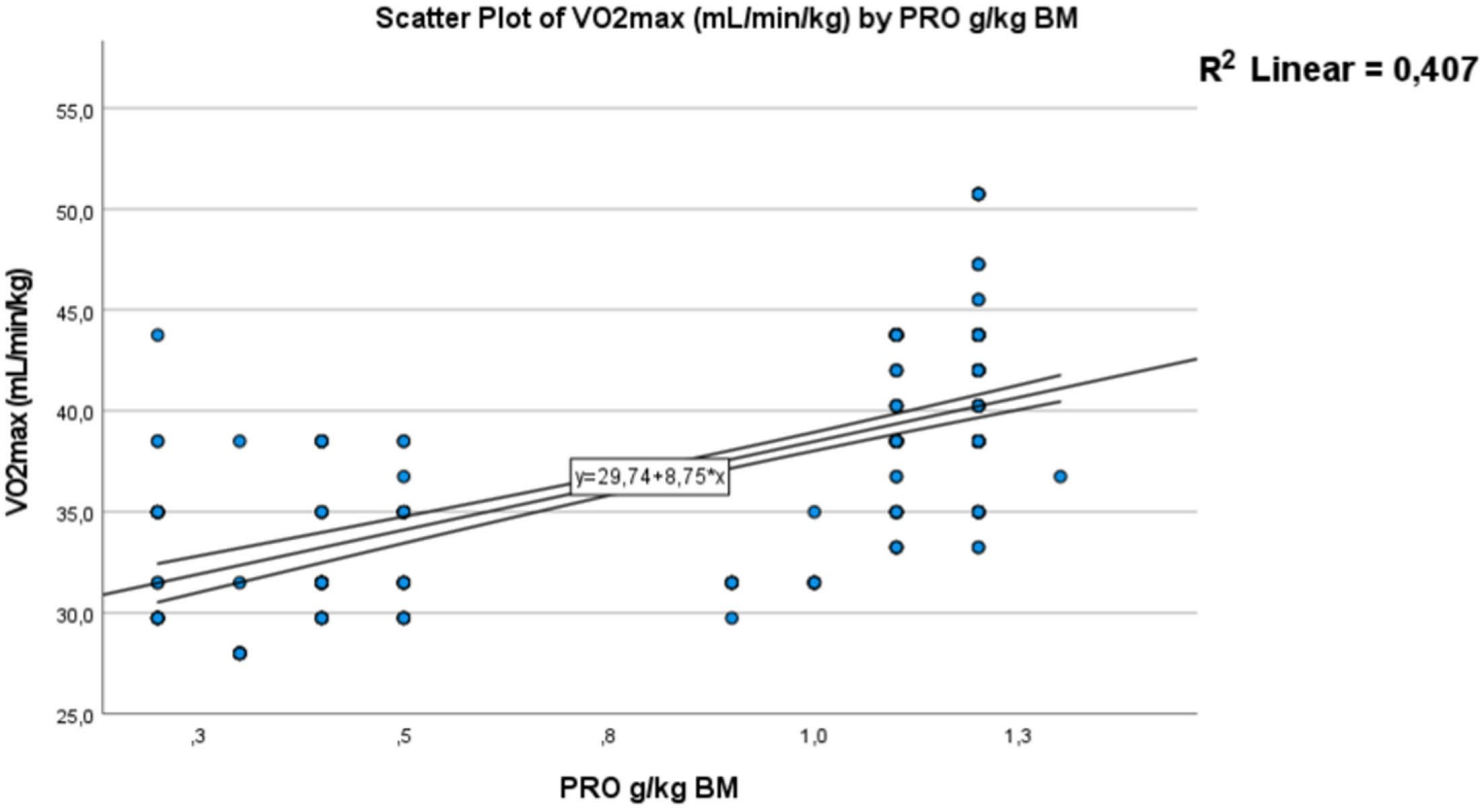
Correlation between the protein intake and Vo_2max_. The dots represent individual participants (*n* = 300). The area is for the 95% confidence interval.

**Figure 12:**
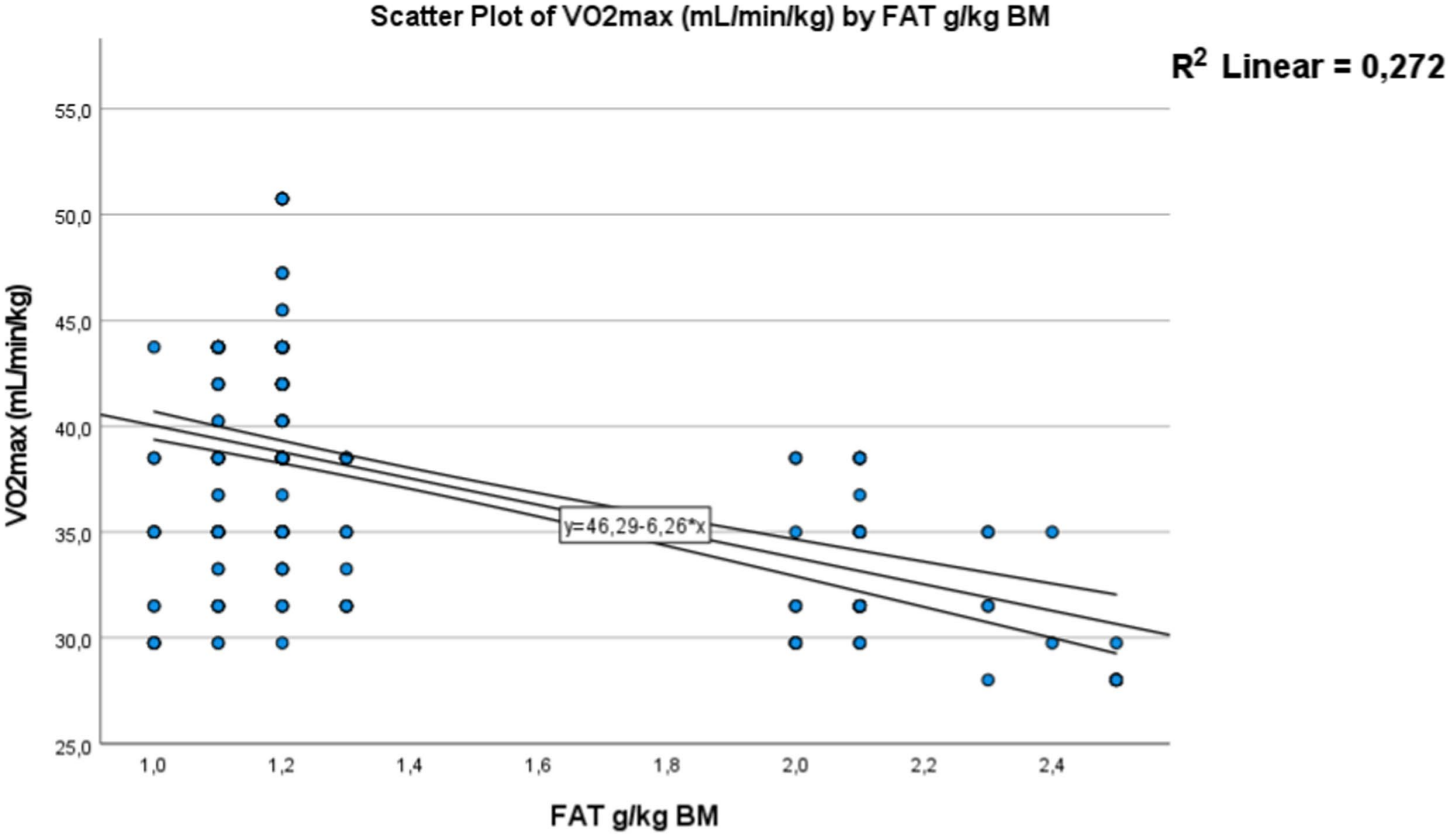
Correlation between the fat intake and Vo_2max_. The dots represent individual participants (*n* = 301). The area is for the 95% confidence interval.

A strong positive correlation was found for carbohydrate intake consumed by adolescents, expressed in grams per kilogram of body weight per day Figure 10 with a *p*-value <0.01, a correlation coefficient (*R*) of 0.69, and a coefficient of determination (*R*^2^) of 0.476. Adolescents who adhere more closely to the recommended carbohydrate intake tend to have better physical fitness levels and the necessary energy to perform well in the Cooper test, covering a greater distance. This positive relationship is also observed for protein intake Figure 11. Where a strong significant correlation was recorded, with a *p*-value <0.01, a correlation coefficient (*R*) of 0.638, and a coefficient of determination (*R*^2^) of 0.407. Adolescents with protein intake within the recommended guidelines have greater muscular capacity to support exercise. Conversely, higher levels of fat consumption hinder achieving positive performance results Figure 12. A strong negative correlation was clearly detected, with a *p*-value < 0.01, a correlation coefficient (*R*) of −0.52, and a coefficient of determination (*R*^2^) of 0.272. As fat consumption increases, performance decreases. Also Based on these all results, hypothesis 2 (H2) has been confirmed.

##### 3.4.3.2. Correlation between Vo_2max_ and protein in g/kg of body mass in day

Correlation between the protein intake and Vo_2max_. The dots represent individual participants (*n* = 300). The area is for the 95% confidence interval as shown in Figure 11.

##### 3.4.3.3. Correlation between Vo_2max_ and fat in g/kg of body mass in day

Correlation between the fat intake and Vo_2max_. The dots represent individual participants (*n* = 301). The area is for the 95% confidence interval as shown in Figure 12.

## 4. Discussion

### 4.1. Physical fitness is significantly associated with the status of macronutrient intake

The aim of the study was to measure the influence of macronutrient intake and body composition on physical fitness. This study demonstrated that nutritional status is significantly associated with physical fitness (PF) levels in both males and females. Dietary energy intake was considered insufficient compared to the recommended levels. And the adolescents who adhere more closely to the recommended macronutrient intake tend to have better physical fitness levels.

The nutritional status exhibited a direct correlation with physical fitness (PF), as assessed by cardiorespiratory endurance through a running test (48), and an indirect correlation with aerobic capacity (49). Moreover, a more balanced nutritional status in terms of energy and macronutrients, particularly carbohydrates and proteins, coupled with reduced fat intake, was associated with higher levels of physical fitness (PF). Conversely, an imbalanced nutritional status showed a negative relationship with physical fitness (PF). According to the study, these young students have an average dietary energy intake of 2,387 ± 492 Kcal, which is considered insufficient compared to the recommended levels. Furthermore, most girls have an energy intake below the recommended levels to meet the demands of this physical effort (*t*-test <0.01). On other hand, boys have a higher energy intake of 2,486 ± 531 Kcal compared to girls, who have a 2304.0 ± 437 Kcal. Additionally, the proportion of energy from fat intake is significantly higher in girls (34.0% ± 10.9) compared to boys (30.0% ± 10.9) (*p*-value < 0.01).

This association confirmed in this study between that nutritional status and physical fitness was represented by the strong positive correlation found for carbohydrate intake consumed expressed in grams per kilogram of body weight per day and Vo_2max_ (*p*-value < 0.01, *R* = 0.69, and *R*^2^ = 0.476). Adolescents who adhere more closely to the recommended carbohydrate intake tend to have better physical fitness levels and the necessary energy to perform well in the mini-Cooper test, and covering a greater distance. A similar positive relationship was observed for protein (*p*-value < 0.01, *p*-value, *R* = 0.638, *R*^2^ = 0.407). Adolescents who consume protein within recommended guidelines tend to have greater muscular capacity, supporting improved exercise performance. Conversely, higher levels of fat consumption hinder achieving positive performance. A strong negative correlation was clearly detected (*p*-value < 0.01, *R* = −0.52, *R*^2^ = 0.272). As fat consumption increases, performance decreases.

Our findings support the current studies for this age group. An epidemiological cross-sectional study called HELENA was conducted in Europe from 2006 to 2007, involving over 3,500 adolescents aged 12.5 to 17.5 years residing in 10 cities across 9 European countries. Its objective was to assess the comprehensive nutritional status of these adolescents, including body composition, biology, physical activity, and physical fitness. The results of this extensive study reveal the influence of numerous individuals, collective, and environmental factors on the nutritional status, level of activity, and physical fitness of adolescents. Most girls have an energy intake below the range presented in the European Report on Nutrition and Health, which is 2,270 to 3,470 kcal/day for boys and 1,630 to 2,320 kcal/day for girls (54). In a recent study conducted in Casablanca, Morocco in 2022, on a similar population over the course of 1 week, it was observed that adolescents did not meet the recommended daily energy intake. The average daily caloric intake was 2134.7 ± 1.4 kcal, with a macronutrient distribution of 53% carbohydrates, 13% proteins, and 34% fats (55). A research study conducted in China in 2020 confirms the positive association observed between nutritional status and the level of physical fitness (56) also a retrospective study was based on data from the “Observatory of French physical fitness^©^” using the physical tests included in the Diagnoform Tonic^©^” battery, Showed that boys had better cardiorespiratory fitness than girls during adolescence (57).

The difference in physical fitness level between the two sexes can be explained by the availability of energy and the metabolic demands of physical exercise (58). During physical exercise, such as in the case of fitness test, numerous biochemical and biological changes occur in the body to meet increased demands for energy and oxygen. Firstly, cellular energy metabolism, especially in muscle and liver cells, is affected. Physical exercise stimulates an increase in energy demand for muscle contraction, leading to elevated glucose and fatty acid breakdown to produce adenosine triphosphate (ATP), the primary source of energy used by cells. Several metabolic processes are activated to generate ATP, including glycolysis, fatty acid beta-oxidation, and the Krebs cycle (59, 60). These metabolic processes enable the production of ATP necessary to support muscle activity during physical exercise. Given that adolescents have an adequate intake of energy and proteins, they achieved better performance in the test compared to others.

### 4.2. Physical fitness is significantly associated with the body composition

This study confirmed that body composition significantly affected the physical fitness and, in both males, and females. As compared to the obese and overweight categories according to the BMI, significantly higher scores of physical fitness PF were observed for males and females in the normal-weight group. The results suggested that students in the normal category indicated greater physical fitness and good physical health; physical fitness became better and then worse with increased BMI. According to the findings, and considering age, gender, and recommendations, a large portion of the study population exhibits an unbalanced dietary behavior, characterized by a tendency towards Fat-rich food consumption *p*-value < 0.01, especially among girls. This observation was further supported by the assessment of weight status, which revealed a higher prevalence of obesity and overweight among girls compared to boys 16.61% are overweight (10.30% of girls and 6.31% of boys), and obesity is present in 5.32% of the students (3.65% of girls and 1.66% of boys). Our findings are also in line with other internationals researches. In Lithuania, the prevalence of overweight was less than 5%, with rates of 1.8% among 13-year-old boys, 2.6% among 13-year-old girls, 0.8% among 15-year-old boys, and 2.1% among 15-year-old girls (61). A study conducted at the European level presents the prevalence of overweight and obesity among children. The results show a tendency for a higher prevalence of overweight among children in Western Europe, especially in Southern Europe (62).

In 2022, a recent retrospective study from France suggested that the prevalence of overweight and obesity was higher among boys than girls. The study also found that participants with a normal weight had the best physical fitness (PF) levels, while those who were obese had the worst PF levels (57). The Aerobic capacity is closely associated with enhanced health and appears to be heavily influenced by both gender and PA level in adolescents. This underscores the significance of sustaining an adequate level of physical activity during the adolescent years. As indicated in the adolescents Norway cross-sectional research study in 2020 (63).

Our study indicates as like (64, 65) that the BMI has an influence on the Physical Fitness (PF) in both males and females. Individuals categorized as obese or overweight based on their BMI tend to have significantly lower PF scores. Therefore, considering PF is essential for assessing the physical fitness of high school students. This study demonstrated a significant and negative correlation between body fat mass in % and physical fitness (PF) (*p*-value <0.01, (*R*) of −0.52), the adolescents who have a higher body fat mass % have a higher ratio of energy intake, suggesting that they tend to consume a more energy-rich diet. Also, the young student with a high level of physical fitness tends to have a normal body weight, while those suffering from obesity generally exhibit a low level of physical fitness. However, this negative correlation indicates that the adolescents consuming higher levels of fats have poorer physical performance. In conclusion the Major results provided support to previously published findings concerning the negative impact of high % Body Fat (BF) on both aerobic and anaerobic fitness related components (66).

The difference in physical fitness level between the two sexes and the all observed parameters can be explained. Firstly, the adolescence is a critical period of growth (67) and bodily development, during which there are significant changes in height and body mass. At the beginning of this phase, boys tend to experience a more pronounced increase in protein accrual, while girls generally exhibit a greater increase in body fat. On average, boys see an increase of 31 kg in lean mass (including muscles) and 3 kg in body fat. For girls, the increase is distributed differently, with a gain of 15 kg in lean mass and 7 kg in body fat. Consequently, caloric needs are higher in boys (68).

The dietary and health habits developed during adolescence will have a direct or indirect impact on health in the short, medium, and long term in adulthood (69). Obesity leads to numerous complications. Apart from metabolic issues such as insulin resistance and diabetes, the most significant complications are related to the effects of obesity on cardiorespiratory function (70).

In this research, we have confirmed and demonstrated that an adequate intake of macronutrients, particularly carbohydrates and proteins, along with a reduction in fat consumption, contributes to promoting a high level of physical fitness. It is also important to note that excessive carbohydrate consumption, especially refined sugars, can be detrimental to health. An excess of carbohydrates can lead to elevated triglyceride levels in the blood, which is a risk factor for cardiovascular diseases, especially in this age group (71–73).

These findings highlight the importance of monitoring both energy intake and body composition in adolescents, as excessive energy consumption associated with an accumulation of body fat can be concerning for their long-term health and well-being, and may be one of the possible causes of weight imbalances such as overweight or obesity (70).

Due to the specificities of this period, it is advisable to ensure a balanced protein intake to promote growth and bodily development. It is also recommended to diversify food sources, including animal sources such as meat, fish, eggs, milk, and dairy products, as well as plant-based sources like bread, cereal-based products, and legumes (74).

## 5. Limitation and perspective

Our study, the first of its kind in Morocco, aims to assess how nutrition, with a particular focus on macronutrient intake, influences anthropometric parameters and the level of physical fitness among young adolescents. The central objective of our study is to investigate the association between a balanced nutritional status and several key parameters. We aim to determine whether individuals with optimal nutritional balance also exhibit a high level of physical fitness and a body mass index (BMI) within the normal range, and conversely, whether an abnormal BMI (underweight, overweight, and obesity) is linked to a lower level of physical fitness and an imbalance in nutritional status.

It’s important to acknowledge several limitations of this study. Firstly, Within the scope of this study, we did not consider factors such as socioeconomic status, parental education level, nutritional knowledge, and dietary patterns, including traditional, Mediterranean, vegetarian, or other types. This is because our primary focus was to comprehend the impact of nutritional intake on physical fitness, rather than diagnose the underlying factors of nutritional intake. In this regard, we encourage the scientific community to embark on future research endeavors to explore the influence of nutritional education on physical fitness, as well as to investigate potential associations between the socioeconomic status of adolescents and their parents with both physical fitness levels and body composition. Finally Additional studies involving more extensive sample sizes and participants from varied geographical regions, as well as distinct cohorts (e.g., categorized by age), are required to explore and authenticate the results obtained in this study for future research.

The secondary school system is characterized by a significant academic workload, exposing students to considerable pressure. This academic pressure often coincides with a lack of physical activity, as students devote a substantial amount of time to studying and taking exams. This situation contributes to a high prevalence of obesity and an increase in the number of overweight individuals among students (75). It is imperative for parents, educators, and administrators to take steps to preserve the physical health of students, which also has implications for public health. Parameters such as physical fitness, in conjunction with body fat percentage, as we have verified and demonstrated, serve as critical indicators. They are closely linked to various aspects of health, including obesity, cardiovascular diseases, respiratory health, air pollution-related issues, mental health, and facets of social psychology (28, 76).

Furthermore, the environment plays a significant role, as highlighted during the COVID-19 pandemic-related lockdowns (26, 27, 29). All these factors underscore the importance of promoting good physical fitness among students and considering their environment to ensure their overall well-being.

## 6. Conclusion

In summary, Adolescents with BMI values outside the normal range generally exhibit lower physical fitness levels compared to those with normal BMI values. The relationship between physical fitness and BMI, as well as body fat mass, is more pronounced among female adolescents than their male counterparts. Consequently, cardiorespiratory endurance is closely associated with the macronutrient intake profile and, by extension, the dietary behavior of adolescents.

It is worth noting that adolescents who adhere to the recommended levels of carbohydrate and protein intake appear to benefit from enhanced physical fitness, which plays a pivotal role in their overall health and physical performance. Conversely, an excessive consumption of dietary fats may have adverse effects on their overall physical performance.

In this context, there is a pressing need for nutritional education. Furthermore, it is essential to implement strategies aimed at encouraging students to maintain a balanced diet, engage in regular physical exercise, and enhance the quality of physical education.

## Data availability statement

The original contributions presented in the study are included in the article/supplementary material, further inquiries can be directed to the corresponding author.

## Ethics statement

The studies involving humans were approved by the regional ethics committee of the Ibn ROCHD University Hospital Center in Casablanca, Ministry of Health Morocco (Approval No. 22/2022). The studies were conducted in accordance with the local legislation and institutional requirements. Written informed consent for participation in this study was provided by the participants’ legal guardians/next of kin. Written informed consent was obtained from the individual(s), and minor(s)' legal guardian/next of kin, for the publication of any potentially identifiable images or data included in this article.

## Author contributions

MO: Conceptualization, Data curation, Investigation, Methodology, Writing – original draft, Writing – review & editing. KB: Writing – original draft, Writing – review & editing. KM: Conceptualization, Data curation, Investigation, Methodology, Supervision, Writing – original draft, Writing – review & editing. HL: Conceptualization, Data curation, Formal analysis, Investigation, Methodology, Supervision, Validation, Writing – original draft, Writing – review & editing. AD: Conceptualization, Data curation, Formal analysis, Investigation, Methodology, Project administration, Supervision, Validation, Writing – original draft, Writing – review & editing. AK: Conceptualization, Data curation, Formal analysis, Investigation, Methodology, Project administration, Software, Supervision, Validation, Writing – original draft, Writing – review & editing. RS: Conceptualization, Data curation, Formal analysis, Investigation, Methodology, Project administration, Software, Supervision, Validation, Writing – original draft, Writing – review & editing. HT: Conceptualization, Data curation, Formal analysis, Investigation, Methodology, Project administration, Software, Supervision, Validation, Writing – original draft, Writing – review & editing.
